# Perturbation-based Markovian Transmission Model for Probing Allosteric Dynamics of Large Macromolecular Assembling: A Study of GroEL-GroES

**DOI:** 10.1371/journal.pcbi.1000526

**Published:** 2009-10-02

**Authors:** Hsiao-Mei Lu, Jie Liang

**Affiliations:** Department of Bioengineering, University of Illinois at Chicago, Chicago, Illinois, United States of America; Fox Chase Cancer Center, United States of America

## Abstract

Large macromolecular assemblies are often important for biological processes in cells. Allosteric communications between different parts of these molecular machines play critical roles in cellular signaling. Although studies of the topology and fluctuation dynamics of coarse-grained residue networks can yield important insights, they do not provide characterization of the time-dependent dynamic behavior of these macromolecular assemblies. Here we develop a novel approach called Perturbation-based Markovian Transmission (PMT) model to study globally the dynamic responses of the macromolecular assemblies. By monitoring simultaneous responses of all residues (>8,000) across many (>6) decades of time spanning from the initial perturbation until reaching equilibrium using a Krylov subspace projection method, we show that this approach can yield rich information. With criteria based on quantitative measurements of relaxation half-time, flow amplitude change, and oscillation dynamics, this approach can identify pivot residues that are important for macromolecular movement, messenger residues that are key to signal mediating, and anchor residues important for binding interactions. Based on a detailed analysis of the GroEL-GroES chaperone system, we found that our predictions have an accuracy of 71–84% judged by independent experimental studies reported in the literature. This approach is general and can be applied to other large macromolecular machineries such as the virus capsid and ribosomal complex.

## Author Summary

Biological processes in a cell often require complex molecular machineries with large macromolecular assemblies as components. An example is the chaperone system in the bacterium *E. coli*, which helps proteins to fold correctly. In these macromolecular machineries, signals are transmitted dynamically in order for biological functions to be carried out. Studying the dynamic process of signal transmission helps us to identify key elements of the macromolecular assemblies that are pivots for dynamic motions, communicators for interfacing with other molecules, and anchors that are key for signal transmission. In this study, we describe a novel computational method that can globally survey the dynamic responses of the macromolecular machinery to perturbation over the full time course by monitoring simultaneously all the elements at the amino acid residue level and at multiple time spans, from the initial perturbation until the system reaches equilibrium. We show that the key residues predicted by our computational method in the chaperone system of *E. coli* to a large extent are correct, as they often coincide with the ones identified by experimental studies. We also show that this computational method can make novel predictions about the importance of additional amino acid residues previously uncharacterized, which can be further tested in experimental studies. This approach can be applied to study other large macromolecular assemblies such as the virus capsid and ribosomal complex.

## Introduction

In the cellular environment, biological processes often involve large assemblies of macromolecules, such as ribosome complex in protein synthesis, assembly of virus capsid, and chaperone systems assisting protein folding. Understanding how signaling communications are transmitted and the dynamics of such allosteric communications in these biological nanomachines at multiple time scale is an important problem.

To explore the roles of large-scale dynamics fluctuations of proteins, coarse grained models such as Gaussian network models (GNM) and elastic network models (ENM) have been developed and are now widely used [1]–[13]. These models are based on a simplified representation of protein structures. The eigen modes of these models are then used to explore the fluctuation dynamics of proteins around their native conformations. Chennubhotla and Bahar have further extended the network model by introducing a Markovian process to describe how signal propagates in proteins [8]. In another study, by clustering residues into groups based on simplifications of the Markovian transition matrix, models of allosteric communication paths can be constructed [8]. By examining pair correlation between residues at slow eigen modes, clusters of coupled residues in potassium channel that are important for inter-subunit cooperativity are identified [14]. Recent work based on topological analysis of shortest paths of network models showed that residues mediating signaling in proteins and modules of protein architecture can be identified [15],[16].

A drawback of current coarse grain network model based studies is that they do not characterize the time-dependent behavior of dynamic changes in protein assemblies. Furthermore, they often require additional mode analysis to assess the relevance of fluctuations associated with individual eigen mode [8]. Although the dominant modes most important for biological functions are often understood to be the slow modes [7],[8], their precise identification often cannot be determined *a priori* and requires significant amount of analysis with additional experimental information [10]. Frequently, the most important mode is different for different proteins. A recent study by Zheng, Brooks, and Thirumalai showed that by locating robustly conserved residues, evolutionary analysis of proteins through multiple sequence alignment can be helpful in identifying important dynamic modes [10].

In this study, we postulate that rich information about allosteric dynamics relevant for biological function can be directly obtained from native structures without any mode analysis or multiple sequence alignment. Our approach builds upon the Markovian stochastic model introduced by Chennubhotla and Bahar [8], and our earlier preliminary work [17]. Chennubhotla and Bahar have firmly established the connection between the Markovian transmission model and the physical elastic network model through the mapping between Markovian hitting time and the equilibrium fluctuation dynamics of residues [18]. Our aim here is different, and it is to study how different parts of a macromolecular machinery respond to signal perturbation. Here the perturbation is given as the initial condition, and our conjecture is that the dynamic response of residues can reveal biologically important information. Our approach is termed as the Perturbation-based Markovian Transmission (PMT) model.

The main task of our study is to characterize the temporal dynamic response to signal perturbation across all residues in a large macromolecular assembly at all time scales, *i.e.*, from the beginning perturbation until the equilibrium state is reached. This is a challenging task, as it is difficult to follow the precise time trajectories of several thousand or more residues simultaneously over 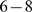 or more decades of time span. For this purpose, we show that using a matrix subspace projection method, the underlying large scale master equation governing the dynamics of Markovian signal propagation can be solved accurately. We also show that simple perturbation can reveal intrinsic dynamic properties of proteins. In addition, we present new results on the characterization of functionally important residues and the mechanism of signal transduction in the large macromolecular assembly of the GroEL-GroES system, which has been studied previously using Gaussian network model [8] and Brownian dynamics simulations [10].

## Results/Discussion

### 

#### Network model for perturbation-based Markovian time-dependent transmission

In our Perturbation-based Markovian Transmission (PMT) model, the dynamic behavior of a large macromolecular complex is probed by applying a perturbation as initial condition, which can be applied to a specific local region of the protein, a subset of surface residues, or all residues in the protein. This perturbation can be regarded as a signal responding to ligand binding or protein-protein interactions. This signal will be transmitted from the location of perturbation to the rest of the macromolecule. Our goal is to study the dynamic process for the time-dependent diffusion of the perturbation signal.

We follow previous studies to model a large macromolecular complex as a network of nodes connected by edges whose architecture solely depends on its three-dimensional structure. In our model, we consider atomic details of contacts between different residues. Details can be found in the **Materials and Methods** section.

#### Markovian model for signal transmission

We use the Markovian transition model [8] to study how a given perturbation is transmitted at different time steps. In each time step of the Markovian process, the perturbation is transmitted from a residue 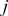 to a neighboring residue 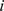 with a probability flow 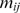. The collection of 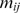 forms the Markovian transition matrix 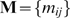. Intuitively, a signal initially placed at residue 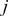 in our model will be completely transmitted to its contacting neighbors within one time step.

We apply a perturbation as an initial signal to probe the behavior of the dynamic response of the macromolecular assembly. Denote the probability of initiating a Markovian process at an individual node 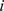 at time 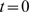 as 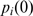. We define the perturbation as the set of probabilities 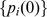. Collectively, we use a vector to denote the distribution of the signal at time 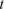 as 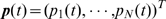 for a system with 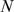 residues, where 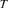 denotes vector transpose. For convenience, we normalize 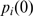 so the probability flow sums up to 1: 
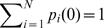
.

#### Time-dependent transmission of perturbation and master equation

The dynamic response of the macromolecular complex in our model is fully determined by the contacts and how signal transmits between contacting nodes. In a Markovian model, the distribution of signal 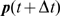 at time 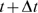 is obtained from the distribution 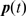 at the previous time step 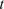 by applying the Markovian matrix 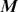: 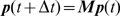. Here 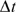 represents the time unit. Repeating this procedure recursively, we have the signal at 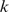 time steps away from 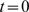 as:
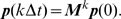



Mathematically, this discrete Markovian process is equivalent to a continuous time process when 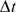 is infinitesimally small, where the change in probability flow at residue 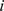 is determined by the amount of the flow entering and leaving residue 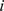:
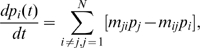
(1)In matrix form, we have the following master equation:
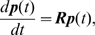
(2)where the rate matrix 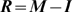, and 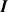 is the identity matrix. The analytical solution of the master equation is
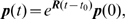
(3)


For a given Markovian matrix 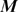, the master equation has an exact solution, which provides an accurate picture of the relaxation process of the initial perturbation in the complex macromolecular system. The final distribution of the PMT depends only on the network structure and can be written in closed-form (see **Materials and Methods**).

#### Dynamic time-dependent evolution of probability flow in Perturbation-based Markovian Transmission (PMT) model

Although the final stationary state depends only on the connectivity of the network and is the same for any arbitrary perturbation, we are interested in the details of the time-dependent dynamic response of the system in response to a specific initial perturbation.

Simultaneously monitoring the exact time evolution of probability flow for all individual residues from the first perturbation until stationary state is a challenging task. Although the probability flow vector of residues at the 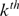 time step can be calculated using straightforward matrix multiplication, this would require 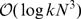 steps of calculation [19], and becomes infeasible for a large system with multiple modes of diverse relaxation times. The analytical solution of the continuous-time version of the model 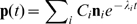 through diagonalization is also impractical, as it is typically only possible to calculate a few eigenvectors and eigenvalues for a very large matrix [19]–[21]. In this study, we use the Krylov subspace method for fast and accurate computation of the time evolution of probability flow [22].

### Chaperone GroEL-GroES Complex

**Figure 1:**
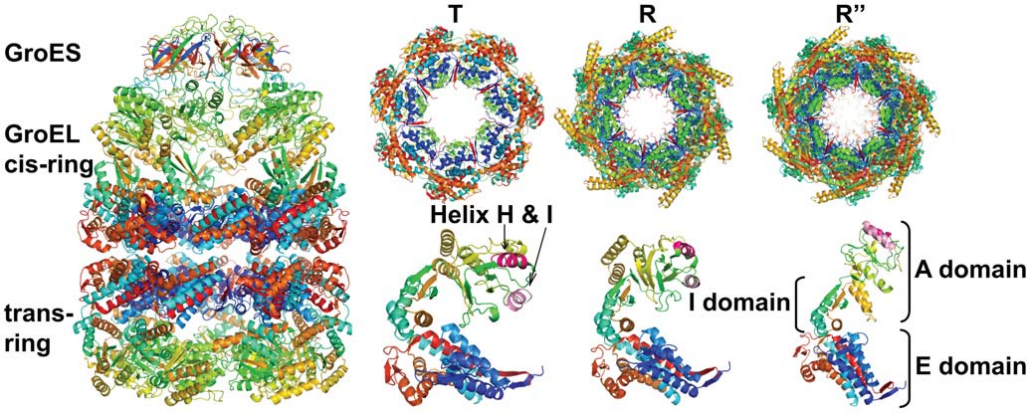
The structure of GroEL-GroES chaperone complex. Side view for the R″ state (left), top view (upper right) and side view (lower right) for the T, R and R″ state (pdb ids 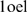, 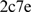 and 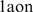, respectively). The GroEL-GroES chaperone complex is large, consisting of 8,015 residues in the resolved structure, with 14 and 7 chains in the homo-oligemeric GroEL and GroES subunits, respectively. Each chain in the GroEL structure contains three domains: the E (equatorial) domain, the I (intermediate) domain, and the A (apical) domain, reflecting their respective spatial positions in the GroEL-GroES chaperone complex.

As an example, we use the PMT model to study the dynamic response of the chaperone complex in *Escherichia coli*. The function of this ATP regulated chaperone complex is to assist the folding of unfolded and misfolded substrate proteins while changing conformations among the T, R, and R″ states (pdb 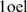, 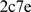 and 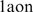, respectively [23]–[25]). The structures of the GroEL-GroES chaperone are shown in Fig 1.

**Figure 2:**
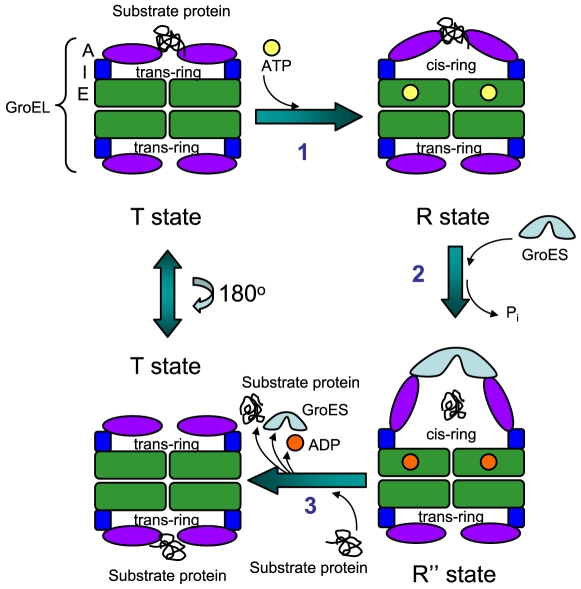
Key conformational states of T, R, and R″ in the simplified allosteric signaling cycle. Each of the 14 identical GroEL chains contains the E domain (equatorial domain, shown in green), I domain (intermediate, blue), and A domain (apical, purple). In step 1 of this illustration of simplified cycle, the A domains in the T state conformation of the GroEL subunits bind a misfolded or unfolded substrate protein. After step 1, GroEL reaches the R state conformation upon binding of 7 ATP molecules on the E domains. In step 2, the A-domain of the ATP-bound form of GroEL binds to GroES, and the substrate protein falls into the central cavity of the chaperone complex. At the same time, ATPs are hydrolyzed to ADP molecules, and the conformation of GroEL-GroES changes to the R″ state. In step 3, a new unfolded protein ligand binds to the other symmetrically related ring of GroEL. Once bound, the GroES, and the now re-folded substrate protein, and 7 ADP molecules are released. The conformation of the GroEL switches back to the T state.

We briefly review our current understanding of the allosteric communications and functional activity of the GroEL-GroES chaperone complex in *E. coli*. The cycle of conformational change starts from the T state, which contains neither the ADP nor the ATP molecules. In addition, the GroES subunit is not yet bound to the GroEL subunit (Fig 2). In step 1 during the transition from the T state to the R state, one of the trans-rings takes seven ATP molecules and interact with the unfolded or misfolded protein substrate. This is followed by conformational changes of the trans-ring to become a cis-ring, which is observed in the R state structure. The ATP molecules in the R state are then hydrolyzed to become ADP molecules. This hydrolysis triggers further conformational change, and the complex transits from the R state to the R″ state in step 2. In the meantime, the GroES subunit binds to the top two helices (H and I) of the cis-ring chains of the GroEL subunit in the R″ state, and the protein substrate is released into the central cavity of the chaperone complex. The last step of the process occurs after a new unfolded protein ligand bind to the original trans-ring chains of the GroEL subunit in the R″ state [26]. The effect is that the cis-ring in the R″ state transits and take the conformation of the trans-ring in the T state, releasing the the GroES subunit, the protein substrate, and the seven ADP molecules during the process. Now the complex goes back to step 1 and ready for another cycle [26],[27].

### Analysis of Dynamic Responses

To understand the dynamic response of the chaperone GroEL-GroES complex, we analyze details of the time trajectories of the residues upon perturbation.

#### Simultaneous time trajectories of perturbation signal

**Figure 3:**
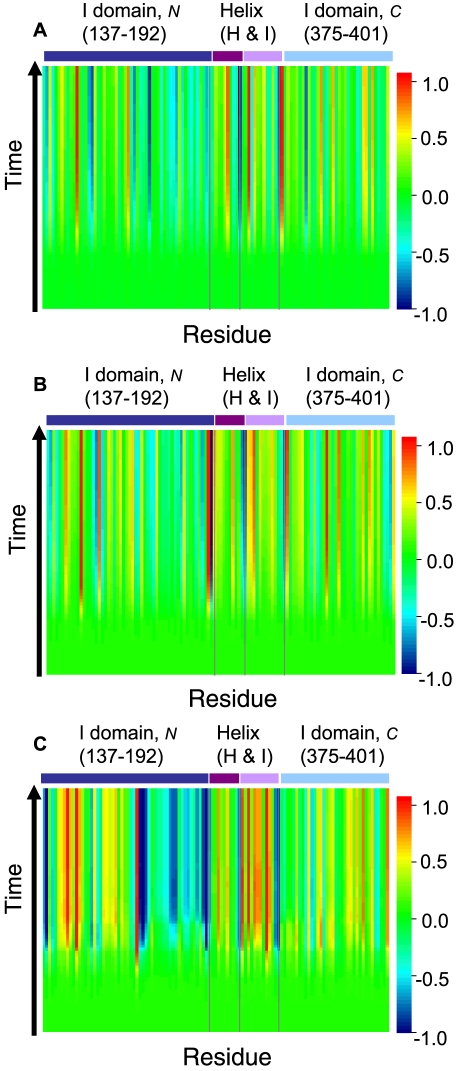
The trajectories of time dependent dynamic responses of residues in the chaperone complex. Responses are calculated using (A) the T state conformation (pdb 1oel), (B) the R state conformation (2c7e), and (C) the R″ state conformation (1aon) of the chaperon complex structures. For clarity, only time trajectories for the N-terminus of the I domain, the helices H and I in the A domain, and the C-terminus of the I domain are plotted. The 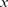-axis represents the numbering of residues in sequence, and the 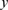 axis indicates the time scale. The value of the probability flow is color coded at different 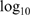 scale. The color coding scheme (shown in the side bar) is derived from a linear transformation of the probability flow: The initial probability value is set at 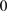, and either the maximum or the minimum among all residues at all time scale, whichever has the largest absolute value, is scaled accordingly to 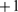 or 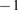.

We apply an initial perturbation of uniform strength to all residues in the macromolecular assembly of chaperone complex and observe the time response of each residues, starting from the initial perturbation until the equilibrium state. An illustration of the time trajectories of response of residues in the N-terminus of I domain, helices H and I in the A domain, and the C-terminus of the I domain of GroEL subunit are displayed in Fig 3 for the T, R, and R″ states.

Initially, all residues start with the same amount of perturbation (colored in green). Overall, residues respond differently to the same uniform perturbation. As time proceeds, some residues experience increase in probability flow, with their trajectories changing color from green to red in Fig 3. Other residues experience overall decline in the perturbation flow, with their color changing from green to blue. There are also residues which experience significant fluctuations in perturbation flow. Finally, there are a substantial number of residues that do not experience much changes in probability flow, with their trajectories staying green throughout the time course. The same residue may respond differently in different state of the protein, as can be seen from the different patterns of helices H and I in the three different states of T, R, and R″ (Fig 3).

#### Relaxation half-time 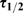


**Figure 4:**
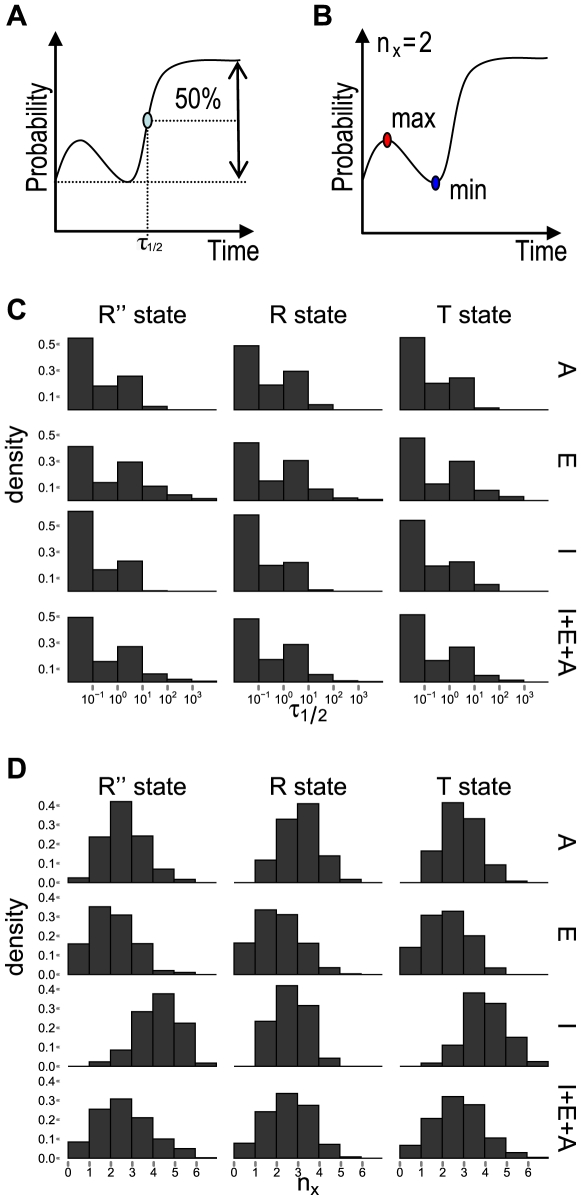
Characterizing the dynamic responses of residues in the GroEL-GroES system. (A) The calculation of the relaxation time 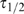. 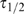 is the time at which the probability flow upon perturbation first reaches 50% of the difference between the initial and the final stationary probability values; (B) The calculation of the number of extrema 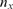. 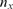 measures the degree of fluctuation in probability flow by counting the total number of local maxima and local minima in the time course of the probability flow; (C) The distributions of the relaxation time 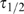 for all residues in the A, E, and I domains of the T, R, and R″ state are shown. Overall, the E domain has the longest/slowest relaxation time, and I domain has the fastest responses; (D) The distributions of the number of extrema 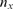 for all residues in the A, E, and I domains of the T, R, and R″ state are shown. Overall, the E domain has the least fluctuations, and the I domain has the greatest amount of fluctuations.

To characterize the overall dynamic response of residues, we define the time it takes for the perturbation signal to reach 50% of the difference between the initial and the stationary probability value for the first time as the *relaxation half-time* 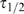 (Fig 4A). It is a simple parameter describing how fast the time dependent response of a particular residue is to the initial perturbation.

Overall, the distribution of the 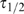 values for different residues span 4–5 orders of magnitude, even though they may all be from the same domain. We find that residues in the E domain have largest overall 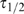 values, with its median values between 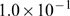 and 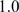 among the T, R, and R″ states, indicating that the E domain has overall the slowest responses to initial perturbation. This is consistent with the fact that the E-domain is the largest domain and its overall relaxation process is slower. Residues in the relatively small I domain have the fastest responses, as the median values of 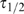 for conformations in the T, R, and R″ states are all in the order of 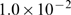. For both E and I domains, the relaxation time of residues does not change significantly among all three states. In contrast, residues in the A domain have overall fast responses in T and R″ states (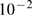), but have overall slower response in the R state (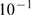) (Fig 4C). This is consistent with the fact the A domain experiences dramatic conformational change between the T and R″ state.

#### Oscillation measured by number of extrema

In addition to the overall patterns of increase or decline in the probability flow, some residues experience significant fluctuation or oscillation in probability flow. We can measure this oscillation with a simple parameter 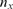, which records the number of extrema points (minimum and maxima) (Fig 4B).

The oscillation number 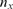 ranges between 0 and 6. Overall, the expected value of 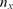 is between 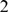 and 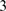, depending on the conformational state (Fig 4D). The distribution of 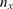 values for all residues in the T, R, and R″ state in the allosteric signaling cycle have overall very similar shape: The histograms shown in the last row of Fig 4D are rather similar among the different conformational states.

However, the domain specific distribution of 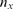 shows different patterns depending on the conformational state (Fig 4D, rows 1–3). This domain-specific distribution changes at different stages of the allosteric signaling cycle (T, R, and R″ states). Residues in the E domain on average experiences least fluctuation in probability flow, and this domain has the smallest median value of 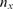 among all domains (between 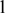 and 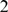, depending on the conformational state, Fig 4D, the 2nd row). In contrast, residues in the I domain (Fig 4D, the 3rd row) on average experiences most fluctuation, with the largest median value of 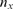 between 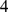 (R″ state) and 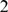 (R state). The largest mean 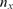 of 4 in the R″ state is related to the significant conformational change the I domain experiences during the allosteric signaling cycle.

In addition, residues of the chaperone complex in the R″ conformation exhibit the largest diversity in fluctuation patterns. In this state, residues in the E domain has a small value of median 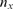 of 1 (Fig 4D, the 2nd row and the first column), whereas residues in the I domain (Fig 4D, the 3rd row and the first column) has a large median value of 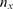 of 4. This is consistent with the fact that the I domain transmits and passes signal between the E and A domain, to which it is connected (Fig 4D).

Overall, there is little correlation between the relaxation time and the number of maxima. The correlation coefficients between 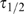 and 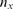 are 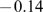 and 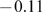, for the T and R conformations, respectively. This correlation reaches 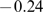 for the R″ state, but is still modest. The general lack of correlation indicates that 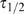 and 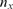 reflect different aspects of the dynamic responses of the protein assembly.

### Characteristic Dynamic Responses of Functionally Important Residues upon Perturbation

We are interested in the response dynamics of residues in the chaperone complex. We describe a few examples of residues experimentally known to be functionally important.

#### Pivot residues

During conformational transitions, domains in the chaperone complex experience large movements, which pivot on several key residues [25]. These pivot residues maintain their spatial positions, while their neighbors experience large spatial displacement. We examine the dynamic response of two known pivot residues, P137 and G192 [25].

P137 is part of the slender link connecting the I-domain and the E-domain. It serves as a pivot for the I-domain, which swings down towards the E-domain and the central channel by *ca*. 25 degrees [25]. When its immediate neighbor C138 with small side chain is replaced by a W residue, which has a bulky side chain, the mutant C138W lost its chaperone function [28]. Pivot residue G192 is located in the slender link between the A- and the I-domains. It is a pivot for a different rearrangement, in which the A-domain swings away from the I-domain by *ca*. 60 degrees, with rotation around its main axis by about 90 degrees [25].

**Figure 5:**
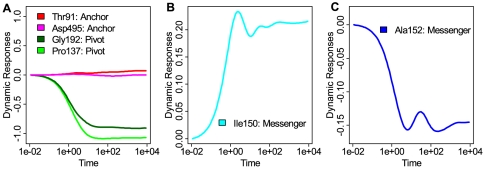
Characteristics of time-dependent dynamic responses of probability flow. Examples of different dynamic responses upon uniform perturbation for residues that have been experimentally determined to be important. (A) Patterns of time-dependent dynamic responses of pivot residues and anchor residues. Responses of the two pivot residues G192 (dark green) and P137 (green) in the T state are smooth and reach low values in the final stationary state. Responses of the two anchor residues T91 (red) and D495 (magenta) in the R″ state have minimal responses to the initial perturbation; (B and C) The dynamic responses of messenger residues I150 (B, cyan) in the T state and A152 (C, blue) in the R″ state. These residues transmit perturbation signals, and experience significant amount of oscillations in the PMT model.

The dynamic responses of these two pivot residues reveal a common pattern. Their probability flows experience smooth changes and eventually reach very low values in their final stationary state, without any intermediate stage of oscillation (Fig 5A). We generalize this observation, and call all residues exhibiting the same behavior in dynamic response to the initial perturbation as “pivot residues”. That is, these residues lose probability flow monotonically with time, until the stationary state of low probability flow is reached. These residues are unlikely to move around during the relaxation process.

Note that residues G375 and G410 reported as pivots in ref [25] do not have the same dynamic responses as that of the pivot residues P137 and G192 (see Supporting Information Text S1 for their characteristic dynamic responses and their functional roles).

#### Messenger residues

The mutations of I150E, S151V, and A152E are known to lead to loss of ATPase activity and loss of GroES binding ability for GroEL [29]. Structurally, S151 forms a hydrogen bond with D87, which interacts Mg^2+^ ion in the ADP binding pocket that coordinates the hydrolysis of ATP to ADP in the R″ state [25]. The mutations of residues near S151 are likely to perturb the R87-Mg^2+^ interactions, resulting in the observed loss of ATPase activity. Without the conversion of ATP to ADP, GroEL cannot change conformations from the R to R″ state, resulting in an arrested allosteric signal transmission.

The temporal dynamic responses of residues I150 and A152 show characteristic patterns of significant periodic fluctuations (Fig 5B and 5C). That is, the probability flows of these residues rise and decline periodically for a few cycles, reaching local maximum and local minimum during the process. This oscillating pattern is different from that of the pivot residues, in which the probability flows decline monotonically (Fig 5A). This oscillating pattern is indicative of their roles in the transmission of signals between different parts of the chaperone. Note that in Fig 5B the response contains both a fast fluctuating high frequency component, and a slow rising low frequency component. The amount of the oscillation can be measured quantitatively using the total number of extrema 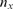 in the time trajectories. 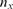 for I150 and A152 are 6 and 5 in the T and R″ state and are among the few residues experience the larger amount of oscillations. We generalize this observation, and call residues exhibiting significant oscillation in temporal response of probability flow to initial perturbation “messenger residues”. The rationale is that these residues pass along signal in probability flow of high frequency to their neighbors, but are affected little themselves by these fast fluctuations.

#### Anchor residues

In the R″ state, residues T91 and D495 are located on the surface of the nucleotide binding pocket in each chain of GroEL, and play important roles in ADP binding by forming hydrogen bonds with ADP [25]. T91 is a hydrogen bond donor, and D495 is a hydrogen bond acceptor in the R″ state [25]. These functionally important residues also exhibit characteristic patterns in their dynamic response to the uniform perturbation: Both experience minimal changes in the probability flow upon perturbation throughout the whole time course (Fig 5A). We generalize this observation, and call residues exhibiting minimal changes in probability flow in temporal response to initial perturbation “anchor residues”. The rationale is that these residues behave like anchors and always maintain the same level of probability flow. They are minimally affected by signals from their neighbors.

### Predicting Pivot, Messenger, and Anchor Residues

Pivot, messenger, and anchor residues have distinct patterns in dynamic responses to perturbation. These patterns are different and are mutually exclusive. A residue can be either a pivot, a messenger, or an anchor residue in a specific conformational state, but the same residue cannot have two or more different patterns of dynamic responses simultaneously in the same state.

We postulate that additional functionally important residues can be identified through analysis of their dynamic responses upon perturbation. Below we describe the prediction criteria and additional residues predicted to play functionally important roles. In the majority of the cases, our predictions are supported by experimental studies. A complete list of identified pivot, messenger, and anchor residues can be found in Table 1, Table 2, and Table 3, respectively, along with corresponding experimental evidence supporting their roles in the function of GroEL-GroES. Details can be found in Supporting Information (Text S1).

**Table 1:** The list of predicted pivot residues, along with the domains, conformational states, and supporting experimental evidence.

Residue	Domain	State	Possible structural and functional roles
G110	E	T, R, R″	inter-ring contact [25],[30], movement upon ATP hydrolysis [30]
G415	E	T, R, R″	helical turn [25], H-bond with ATP [25]
G431	E	T, R, R″	inter-ring contact [30]
G137	I	T, R, R″	inter-domain pivot residue [25]
G459	E	R″	folding and release of substrate protein [29], inter-ring interaction [30],[33]
G32	E	R	helical turn, H-bond with ADP [25]
S139	I	R	folding of substrate protein [28]
T181, G182	I	R	helical turn, inter-domain contacts [10],[29]
A384	I	R	salt bridge [11],[36], ATPase activity, GroES binding [29]
A243, G244	A	R	salt-bridge [11], binding substrate protein [43]
G306	A	R	salt-bridge [11], inter-subunit interaction [10]
S43, F44	E	T	inter-subunit hydrogen bond [23],[32],[37]
G86	E	T	ATP hydrolysis [25]
G269	A	T	substrate protein binding [43]

**Table 2:** The list of predicted messenger residues, along with the domains, conformational states, and supporting experimental evidence.

Residue	Domain	State	Possible structural and functional roles
K245	A	R″	salt-bridge [11], binding of substrate protein [43]
V273	A	R″	binding of substrate protein [43]
G148, A152–S154	I	R″,T	ATPase activity [25],[29], GroES binding ability [29]
D155–T157	I	R″,T	GroEL inter-subunits interactions [38],[39]
V396, L400	I	R″	ATPase activity [25]
A406–E408	I	R″	ATPase activity [29], salt-bridge between inter-subunits [35]
T149–S151	I	T	ATPase activity [25],[29], GroES binding ability [29]
K380, T385	A	T	ATPase activity [29], GroES binding ability [29], folding of substrate protein [29], salt-bridge in T and R state [11],[36], GroEL inter-subunits contacts [32]
A399	A	T	ATP hydrolysis [25]
V411	E	R	GroEL inter-subunits interactions [35]
E460	E	R	folding and releasing of substrate protein [29], GroEL subunits inter-ring contacts [32]
R197	A	R	GroEL inter-subunits contacts [32]
N206	A	R	GroES binding ability [29]
N265, T266	A	R	GroES binding ability [29], folding of substrate protein [29]
I270	A	R	binding of substrate protein [43]
V276	A	R	ATPase activity [29]
F281	A	R	ATPase activity [29], folding of substrate protein [29]
I353	A	R	inter-subunit interaction [10]
S358	A	R	salt-bridge [11], GroEL inter-subunits contacts [32]

**Table 3:** The list of predicted anchor residues, along with the domains, conformational states, and supporting experimental evidence.

Residue	Domain	State	Possible structural and functional roles
V27	E	T	assisting substrate protein folding [29]
T30	E	T	ATP binding [25]
A57	E	T, R, R″	allosteric communication [37]
T90, T91	E	T	H-bond to ADP [25]
P462, V464	E	T, R	folding and releasing of substrate protein [29], inter-ring contact [30],[33]
T482	E	T, R	ATP binding pocket [25]
P496	E	T	ATP binding pocket [25]
T517	E	T	inter-subunit contact [30],[34]
A152, V381	I	T	ATPase activity [29], GroES binding ability [29], folding of substrate protein [29]
L400	I	T	ATP hydrolysis [25]
V407	I	T	ATPase activity [29], GroES binding ability [29], folding and binding of substrate protein [29]
E232	A	T	ATPase activity [29], GroES binding ability [29], folding and binding of substrate protein [43]
A258	A	T	ATPase activity [36], H-bond to substrate protein [43], GroES binding ability [29],[43], folding and binding of substrate protein [29],[43]
L262, V263	A	T	binding of substrate protein [29], hydrophobic interaction to substrate protein [43]
T89	E	R	H-bond to ADP in R″ state [25], ATPase activity [29], GroES binding ability [29], folding of substrate protein [29]
A405	E	R, R″	ATPase activity [29], folding and releasing of substrate protein [29]
A466	E	R	inter-ring contact [33]
V499, V510, A511	E	R	salt-bridge [35], inter-subunits interaction [35]
I150	I	R	ATPase activity [29], GroES binding ability [29], folding and releasing of substrate protein [29]
L200	A	R	GroES binding ability [29], binding and folding of substrate protein [29],[43]
I230	A	R	interaction of substrate protein [43]
V273	A	R	binding of substrate protein [43]
S79, A81	E	R″	salt-bridge [24]
I493–D495	E	R″	ATP binding pocket [25], H-bond to ADP [25]
C519	E	R″	inter-subunit contact [30], releasing of the substrate protein [34]
I205, N206	A	R″	GroES binding ability [29], binding of substrate protein [29]
D328	A	R″	GroEL inter-subunit interaction [37]

#### Predicted pivot residues

We identify pivot residues by the criteria that they experience large declines in the probability flow between the initial state and the final stationary state, without transient increase along the full time course. We select the top 5% out of 524 residues that follow these patterns in the chaperone structure of different states. All together, we have identified twenty-four residues as pivot residues. All of them are found to be located at helical turns. These residues likely serve as hinge residues during conformational changes. For many of them (about 71%), there are experimental evidence and computational studies indicating their important structural or functional roles, such as involvement in salt-bridge interaction, inter-subunit hydrogen bonding, inter-ring contact, ATP hydrolysis, substrate-protein binding, folding, and release (Table 1 and Supporting Information Text S1).

#### Predicted messenger residues

To identify messenger residues that may facilitate communications between different parts of the protein, we select the top residues with the largest number of extrema (no less than that of the residue at 95 percentile in the combined number of minima and maxima) in their time trajectories of probability flow. All together, we have predicted 62 messenger residues in the cis-ring chains of GroEL from conformations in the T, R, and R″ states. Some of the residues are adjacent to each other and are predicted as clusters of messenger residues. These clusters of messengers occur in T and R″ states, indicating the important roles they play in allosteric communication of signal propagation. Overall, 50% among those predicted messenger residues have clear supporting evidence from either biochemical experimental studies or structural data. For example, many residues are known to be involved in salt-bridge, ATPase activity, GroES binding ability, substrate protein binding, or in assisting substrate protein folding, and inter-subunit interaction (Table 2 and Supporting Information Text S1).

#### Predicted anchor residues

These residues respond little to the initial perturbation throughout the time course. They experience minimal changes in the time-dependent probability flow. Sorting residues by the difference of the maximum and the minimum of the probability flow in ascending order, we select the top 5% of residues in the cis-ring chains of GroEL in the T, R, and R″ state with the smallest difference. Among the 68 predicted anchor residues, 54% of them have clear supporting evidence from either biochemical experimental studies or structural information (Table 3 and Supporting Information Text S1).

#### Success rate in identifying functionally important residues

Based on comparison with known experimental and structural data, we calculate the success rate of our predictions. If a predicted residues is within 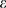 residues apart in the primary sequence from a key functional residue reported in the literature, where 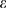 is the sequence distance threshold, we consider it a success. We used sensitivity, specificity, and accuracy to measure the success rate (see **Materials and Methods**). Since there is a large body of experimental studies on GroEL subunits and less on GroES subunits, we assess our predictions using GroEL. At the threshold of 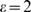, the sensitivity, specificity and accuracy of our predictions on GroEL subunits are 93.9%, 83.4% and 84.4%, respectively. Tables 1, 2, and 3 contains a complete list of the successfully identified residues, and Table 4 contains the list of miss-classified residues at different thresholds. The complete prediction and justification are listed in Table 1,2 and 3 and in Supporting Information Text S1.

**Table 4:** Evaluation of predictions supported by experimental data at different threshold 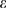 level.

Threshold level 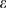	Sensitivity (%)	Specificity (%)	Accuracy (%)	Number of missed residues (missed residues index)
0	42.9	73.7	70.8	28 (R58, K80, D87, A109, C138, Y199, Y203, F204, L234, L237, E238, A241, E257, L259, A260, T261, V264, R268, V271, D383, K386, D398, E409, E461, S463, N479, A480, R501)
1	81.6	79.2	79.4	9 (Y203, L234, L237, E238, A241, A260, N479, A480, R501)
2	93.9	83.4	84.4	3 (L237, E238, N479)

The threshold 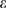 represents the allowed separation distance in number of residues along the primary sequence between predicted and reported residues. The predicted residues are for all three states and include pivot, messenger, and anchor residues.

Although about 1/5 of residues are identified as functionally important, we conjecture that the most critical regions of the proteins have residues predicted to be pivot, messenger, or anchors in multiple conformations. For example, the important interaction between the two GroEL rings are carried out through the movement of helix D (residues 89–109) on the E domain and the inter-ring contacts (A109∶A109, E434∶E434, G461∶R452, S463∶S463, and V464∶V464). Our predictions identified a set of critically important residues from a local region based on perturbation studies of multiple conformations. It includes residues T89-T91, G110, G431, G459, E460, P462, V464, and A466. All of them come from the contact interface between the two GroES rings, and the helix D whose movement is critical for inter-ring communications [25],[30].

To sum up, our results show that many of our predictions of pivot, messenger, and anchor residues are consistent with experimental conclusion that they are functionally important. In addition, our predictions are often in agreement with results from other computational studies based on different methodologies [8],[10],[11]. For example, residues found computationally in previous studies to be involved in multiple salt-bridge switches [11], residues participating in inter-subunit communication [8], and residues forming contact essential for state transition [10] are also predicted in this study to be important in this study.

#### Functional roles of predicted residues

In order to investigate the specific roles of the residues predicted to be pivots, messengers, and anchors, we broadly classify the experimental data on functions of these residues into three categories: (1) GroEL inter-subunits interactions. These include inter-chain and inter-ring communication, as well as salt-bridge switches that form and break between subunits during the cycles of the T, R, and R″ states; (2) ATPase activities. These include ATP hydrolysis, H-bonding with ATP/ADP, or location on the ATP binding pocket; (3) Interactions with GroES and the substrate protein. These include binding, release of GroES and the substrate protein, and facilitation of the folding of the substrate protein.

With these criteria, there is a clear pattern in which pivot residues dominate in inter-subunit interactions, suggesting that they play important roles in assisting inter-subunit movement. We find that for 12 (71%) out of the 17 predicted pivot residues, there are experimental evidence supporting their roles in GroEL inter-subunit interactions (category 1). As a residue may play multiple roles, some of these residues also participate in ATPase activities, or interact with GroES and substrate protein. However, there are overall only 5 (29%) and 6 (35%) out of the 17 pivot residues involved in ATPase (category 2) and GroES/substrate protein interactions (category 3), respectively.

In contrast to pivot residues, the messenger and anchor residues do not show strong preference to any specific functional role. Out of the 31 predicted messenger residues, 16 (52%), 17 (55%), and 17 (55%) are involved in inter-subunit interactions (category 1), ATPase (category 2), and GroES/substrate protein interactions (category 3). Out of the 37 predicted anchor residues, these numbers are 17 (46%), 12 (32%), and 17 (46%), respectively. That is, both messenger and anchor residues may play multiple functional roles in the GroEL-GroES system. We find that there are 17 (55%) and 11 (30%) predicted messenger and anchor residues, respectively, with more than one functional roles reported in the literature.

When compare predictions based on structures of GroEL/GroES in different states, we find that the same residues are usually predicted as pivots in all three states. However, messenger residues and anchor residues are usually state specific. This suggests that the roles pivot residues play in enabling domain movement is universal at all stages of the GroEL/GroES allosteric signaling cycle, whereas messengers and anchors only play important dynamic roles at specific stages of the cycle.

For example, in the R″ state, the predicted messenger residues V396 and L400 in the I domain are spatially close to residue D398, which interacts with a Mg^2+^ ion in the ADP binding pocket in this state [25]. V396 and L400 are only predicted to be messengers in the R″ state. This is consistent with the fact that ATP hydrolysis, as well as the involvement of their neighbor D398 in ADP binding occur in this state.

Residue S358 is predicted to be a messenger, but only in the R state. Residues S79 and A81 are predicted as anchors in the R″ state only. These residues are all immediate neighbors to a salt bridge formed during the R to R″ transition between residues D359 and K80. It is likely that S358, S79, and A81 may play roles in assisting the formation of the salt bridge. S358, S79, and A81 are not predicted to be functionally important residues in the T state. This is consistent with the fact that this salt bridge is absent during the T to R transition [11].

Chennubhotla and Bahar studied the GroEL-GroES system using a model of Markovian propagation. In this study, a hierarchy of coarse-grained models are constructed to partition the structure into soft local regions. Results are inferred based on this grouping of local cluster of residues. Although these two approaches are different, they generate results that agree in many instances. For example, residues P33, T90, E461, and R197 are found in [8] to have high potential to transmit allosteric signals, whereas our predictions include pivot residue G32, messenger residue E460 and R197, and anchor residues T90 and P462, all are from the same local region as those predicted in [8].

#### Evolution conservation of identified residues

From the evolutionary analysis of Brocchieri and Karlin [31], we found that a total of 65 predicted residues are strongly conserved (with Conservation Index 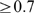 as defined in [31]). Among these, 10 residues (pivot residues G32, G86, G415, and G459, messenger residue G159, and anchor residues T30, V38, T89, D495, and V499) are perfectly conserved (with 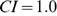). Among our predictions, the fraction of residues with functional roles supported by experimental evidence that are strongly conserved is 41%, 39%, and 41% for pivot, messenger and anchor residues, respectively. Overall, 65 out of the 183 residues we have identified are strongly conserved, representing a fraction of 36%. This does not deviate significantly from random expectation, as there are 183 residues out of a total of 524 residues (35%) in a chain that are found to be strongly conserved in this study [31]. That is, these functional residues are not significantly more conserved than expectation. More detailed evolutionary studies may be necessary to further clarify this issue.

### Interdependency and Cooperativity

#### Interdependency of the two GroEL rings

The interactions of the two GroEL rings depend on between-ring communications, via both direct contacts [30],[32],[33] and through the movement of helix D [25]. A number of residues (T89-T91, G110, G431, G459, E460, P462, V464, and A466) known to be important for cross-ring communications are predicted as pivot, messenger, or anchor residues. These predictions corroborate well with experimental results reported in the literature.

It has been reported that upon ATP binding in the cis-ring, the relay helix D on both cis- and trans-rings move towards the interface between the rings [25]. In addition, helix D in the cis-ring moves towards the inter-ring interface and away from the ATP binding site [30]. Residue G110, located at one end of helix D (residues 89–109), is predicted as a pivot residue in all of the three conformational states of T, R, and R″. This prediction suggests that G110 serves as a hinge that facilitates the physical movement of helix D in all three states. Furthermore, residue A109 in helix D, an immediate neighbor of G110, forms direct inter-ring contact with another A109 located in the other GroEL ring [30]. A109 experiences a large movement (3.8 Å) during cross-ring communication [25]. This movement is pivoted around the predicted pivot residue G110. The functional roles of G110 in facilitating interaction between the cis-ring and trans-ring of GroEL has also been reported in another computational study [10].

Similarly, residue G431 is predicted as a pivot residue in all three states. It is a neighbor of residue E434, which forms inter-ring interaction with another E434 on the other GroEL ring [30], after a large scale structural rearrangement, in which residue E434 experiences one of the largest shift (4.9 Å) [25]. Our results suggest that residue G431, which is predicted as a pivot residue, serves as a hinge to facilitate this large rearrangement. Residues T89-T91, located at the other end of helix D, are predicted to be anchor residues in the T and/or R states (see Table 3 for details). Their roles in cross-ring communications are associated with the large movement of helix D.

Pivot residue G459, messenger residue E460, and anchor residues P462, V464 and A466, are the remaining residues predicted to be involved in ring-communications. They are all located in the neighborhood of several inter-ring contacts (E461∶R452, S463∶S463, and V464∶V464) between residues in the E-domains of different GroEL rings [32]. Mutant E461K no longer forms an inter-ring contact between E461 and R452. Instead, it makes contact with residue E434. This mutant loses the abilities in aiding protein folding and in releasing the substrate protein [29], and has defective activity *in vitro* [30]. In the same neighborhood of the predicted residues, the quadruple mutation R452E/E461A/S463A/V464A is reported to result in the dissociation of the two rings in GroEL [33]. Furthermore, a previous computational study has also suggested that mutations on E461-V464 will hinder signal transmission between rings and destroy the stability of GroEL complex [8].

#### Cooperativity between GroEL subunits

In order to study the cooperativity between different GroEL chains, additional computations based on the structure with all inter-chain contacts removed are performed. Compared to previous results when all contacts are intact, there are six residues (messenger residues R197, I353, S358, T385, and E408, and anchor residues C519) that disappear from the new set of predicted pivot, messenger, and anchor residues. These residues are known to play important roles in the inter-chain allosteric communications [8], [10], [11], [30], [32], [34]–[36].

Similarly, computation based on the modified structure of GroEL with intra chain contacts between different domains further removed are performed to study the cooperativity between intra-chain interactions. We find that additionally the anchor residue D328 disappears from the set of predicted residues. This finding corroborates the fact that residue D328 is known to be involved in one of the important intra-subunit interactions [37]. Our results suggest that the cooperativities of both intra- and inter-chain interactions in GroEL are important for allosteric communications.

The documented roles of these seven residues in transmitting allosteric signals are summarize as below. In the T state conformation, the predicted messenger residue R197 on the A-domain forms a salt-bridge with residue E386 on the I-domain of another chain [32],[36]. This residue likely plays an important role in passing signals between chains [8]. As GroEL proceeds along the allosteric cycle and changes conformation from the T state to the R state, the salt bridge between E386 and R197 breaks up, and residue E386 subsequently forms a new salt-bridge with a different residue K80 on another chain [36]. The formation and breaking-up of the salt bridges provide an important switching mechanism for the allosteric structural changes of GroEL [11].

The predicted messenger residue I353 is a neighbor of residue Q351, which is important for the inter-subunits interactions in the A-domains of cis-ring in GroEL [10]. The predicted messenger S358 is an immediate neighbor of residues D359 and Y360. Y360 and A384 form an inter-subunit contact between the I-domain and the A-domain in the T state [32]. D359 forms a salt bridge with K80 during the R to R″ transition [11]. In addition, the predicted messenger residue E408 is an immediate neighbor of E409. The latter forms a salt-bridge with R501, which is important for inter-subunits interactions [35]. Mutation of E409A results in a stabilized T state relative to the R″ state leads to a less effective allosteric communication in the GroEL complex [35]. C519 is a predicted anchor residue. The mutant C519S destroys the inter-chain contact between the subunits in GroEL, resulting in partial dissociation of the GroEL subunits [30],[34]. The predicted anchor residue D328 is known to be one of the network residues involved in the inter-subunit interaction of GroEL with R58 and A81 [37].

Additional evidence for the positive cooperativity among the seven subunits within each ring has been reported in the literature [38],[39]. It was shown that the single mutation of D155A breaks the interaction between D155 and R395, which in turn destroys the intra-ring symmetry, as less cooperative intermediates are generated that only switches conformations of a subset of the GroEL subunits upon the ATP bindings [38]. Together with the study of unbiased molecular dynamic simulation by Sliozberg and Abrams [39], these studies show that the break of the salt bridge between D155 and R395 from the T state to the R state plays critical role in the positive cooperativity of the concerted allosteric transition. We have correctly predicted messenger residue D155 directly involved in the D155-R395 salt bridge, as well as residues A394 and V396, which are immediate neighbors of residue R395.

### New Predictions of Residues, Pathways, and Testable Hypotheses

#### New predictions and experimentally testable hypotheses

Our study also makes a few new predictions which can be verified by experimental mutation studies and other biochemical studies.

Among the six predicted common pivot residues (G45, G110, P137, G415, G431, and G471) for the T, R, and R″ states in the GroEL subunits, two of them (G45 and G471) have not been experimentally verified as functionally or structurally important. These residues are all located in the E-domain of GroEL. Their functional roles can be further verified by assessing how mutation of these residues may impact the activities of the GroEL-GroES complex. Among the predicted messenger residues, A145, V177, and A402 are found in both T and R″ states. These residues are all on the I-domain of the GroEL subunits, and may play important roles in assisting the allosteric signal passing between the E- and A-domain of GroEL subunits. Further mutational studies of these residues may help to clarify their potential roles on the integrity and activity of the GroEL-GroES system. The discovery of L62, L104, and M491 on the E-domain as potential anchor residues in at least two states may also be considered for further experimental studies.

#### Allosteric signaling transmission pathway

To study the allosteric signaling transmission pathway of the GroEL-GroES complex, we apply a different initial uniform perturbation that is restricted to a set of residues located in the nucleotide binding pocket, and observe the time-dependent dynamic responses of all residues in the chaperone complex. These residues are N479, A480, G415, D495, G32, T91, D398, D87, S151, L31, I454, I150, I493, P33, and A481, which are reported to either form H-bond or to have van der Waals interaction with the ADP molecules [25]. Since there are multiple symmetry related identical chains, we take the average values of the time-dependent probability flow for residues in multiple chains from the same ring.

At each time step, we record the top 3% (16 residues) with the largest probability flow among the total 524 residues in each symmetry related chain in the cis-ring. Accumulatively, there are 28 residues which are observed to have the maximal probability flow one time or another, and they constitute the major components of the time-dependent transmission pathway. These 28 residues are: T30, N68, K75, K80, D83, G86, T90, D115, R118, K132, T149, I270, V276, P279, D291, K327, E354, E397, H401, R404, G414, Q453, Y478, T482, E483, I489, G492, and L494. A movie depicting the process of signal transmission along the pathway is provided in Supporting Information (Video S1). Overall, the perturbation signal at the nucleotide binding site is transmitted from the binding pockets in the E-domains of the GroEL cis-ring towards the GroES chanperonin through the I domains, then the A domains. Among the 28 residues, T149, I270, and V276 are also predicted as messenger residues when a uniform perturbation is applied to all residues in the GroEL-GroES system. These residues are likely to play important roles in general allosteric communication, and specifically in responses to the perturbation signal originating from the nucleotide binding pockets.

We note that these residues do not form a temporally connected pathway, because of the technical criterion of selecting only residues with the maximal probability flow at a time. Frequently, the perturbation signal is transmitted along multiple pathways in a local region of the structure, and the probability flow at these instances is distributed among several residues. As a result, none of these residues will have the maximal flow, and hence are not selected. Overall, the selected 28 residues are representatives of the critical points along the pathway that have the greatest probability flow at a particular instance. Additional residues also contribute to the transmission of the probability flow, but to a lesser extent individually. With the probability flow quantified for each residue at each instance, additional criteria may need to be developed to illustrate other aspects of the allosteric pathways of dynamic responses.

Although the process of the initiation of mechanical conformational changes upon allosteric signaling is not described for explicitly in our model, we do find in some cases that residues transducing allosteric signal are related to pivot residues serving as mechanical hinges during conformational changes. For example, residue G110, located at one end of helix D (residues 89–109), is predicted as a pivot residue in all three states of T, R, and R″. It is a hinge residue that facilitates the physical movement of helix D. Several residues (T30, Q453, K75, K80, D83, G86, T90, D115, and R118) in or near helix D are identified to be on the allosteric signalling pathway, which are spatially close to the pivot residue.

#### Remark

Our approach is based on the Markovian propagation model first developed in reference [8], with some differences in specific definitions of contacts as described in **Materials and Methods**. However, our method is fundamentally different. Perhaps the most significant difference is that our method explicitly computes the dynamic time-dependent responses of residues to an initial perturbation, while the method described in [8] generates information on fluctuation of the stationary state.

In addition, the study of GroEL/GroES system in [8] is based on a hierarchy of simplifications of the contacting nodes and contacting interactions. Coarser grained nodes are used to represent participation of residues to different clusters in the local regions probabilistically. These coarse grained models are derived based on the objective function of minimizing the difference in the stationary distributions between the full residue model and the simplified model. In contrast, no such simplifications are made in our method.

Although it is possible to obtain good agreement in the stationary distribution between the simplified model and the full residue model, these models may still have different dynamic responses, as none of the modes higher than the stationary mode are included in the objective function to be minimized [8]. The Expectation Maximization (EM) algorithm described in [8] will find locally optimal solution to approximate the stationary distribution, but such solution depends on the initial starting point, may not be unique, and may have very different higher-order dynamics.

### Conclusion

In this study, we have developed a new approach, called the Perturbation-based Markovian Transmission (PMT) model, to study the dynamic properties of large macromolecular assemblies. In this model, we probe the macromolecular assembly with an initial perturbation, and assume perturbations are transmitted by a Markovian processes through the connected network defined by the contacts between atoms and residues. This method enables us to monitor the time-dependent responses of all residues simultaneously from the moment of perturbation until all residues reach the equilibrium state. Our method can also be modified to study signal transmission pathways by applying initial perturbation to restricted local region such as binding surfaces. It is effective for very large macromolecular assemblies, requiring relatively small amount of computational time. For example, the computation of probability flows for all 8,000 residues in the GroEL-GroES system takes about 5 minutes on a machine with 2 dual core AMD Opteron processor (1.8 GHz) and 4G memory.

We have applied the PMT model to study the GroEL-GroES protein chaperone system. With an initial *in silico* uniform perturbation to all residues in the T, R, and R″ conformational states, we follow the time-dependent response of 8,000 residues simultaneous until the stationary state is reached after 5 decades in time. Our analysis of the responses of experimentally identified residues known to be functionally important show characteristic patterns. We have further characterized and categorized specific patterns for pivot residues, messenger residues, and anchor residues important for signal propagation in the GroEL-GroES system. Based on these patterns, we have further predicted additional residues that are likely to play important functional roles. Our predictions are largely consistent with experimental results on their roles during the allosteric conformational changes between states. In addition, we have also made a number of novel predictions of functionally important residues, which can be verified by experimental tests. A model of signal transmission pathway has also been developed by applying the intial perturbation to residues in local binding surface.

The successful application of the PMT model to the GroEL-GroES chaperone system for studying its dynamic properties shows that this technique works well. It can be applied to other large macromolecular machineries for detailed computational studies. We envision that the PMT method can be applied to virus capsid, ribosomal complex, and other large allosteric multi-protein assemblies.

## Materials and Methods

### Structural Data of the GroEL-GroES System

The structure of the GroEL-GroES chaperone complex in the T, R, and R″ states are obtained from the Protein Data Bank (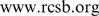). The pdb IDs are 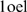, 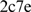 and 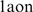, respectively [23]–[25]). The GroEL-GroES complex of the R″ state consists of 14 chains for the homo-oligemeric GroEL subunits, and 7 chains for the GroES subunits, with a total of 21 chains and 8,015 residues. The GroEL subunit structure contains 7 of the 14 chains (3,829 residues) in the T state, and 14 chains (7,658 residues) in the R state (Fig 1).

Since there are multiple symmetrically related identical chains in each ring, we take the average values of the time-dependent probability flow of equivalent residues from different chains located in the same ring.

### Network Model and Signal Transmission

We obtain the contact network of a protein molecule by identify contacts between different residues. Two atoms from different residues are regarded to be in contact if the Euclidean distance between them is 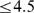 Å. A residue is represented as a node, and an edge between residues exists if they have atomic contacts. The perturbations are signals and can be transmitted from a residue to its neighboring residues in contact with a probability flow following a Markovian processes.

When the perturbation is transmitted from residue 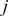 to a neighboring residue 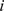, the probability flow 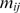 in our PMT model is defined as: 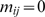 if there is no atom-atom contacts between residue 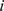 and 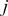, and 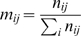 if there is at least one atom-atom contacts between residue 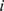 and 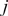. Here 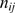 is the number of atom-atom contacts between residue 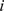 and 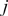. If 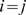, we set 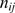 equal to the number of atoms without contact with any atoms from other residues. The Markovian transition matrix 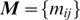 is a stochastic matrix because each column sums up to 1: 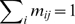.

In our Markovian model, all of the atom-atom contacts of residue 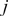 with other residue(s) will transmit the same amount of probability flow. This is different from the model used in reference [8], where the probability flow is not equally distributed among different atom-atom contacts, as the probability flow in that model is weighted by both the size 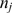 of the source residue 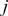 and the size 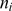 of the target residue 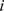: 
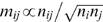
, where 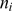 is the number of atoms in residue 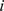.

### Stationary Distribution of Probability

For a Markovian process, the final stationary state distribution 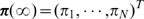 of the signal is independent of the initial perturbation, and has the property: 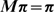. This unique stationary distribution can be written out in closed-form [40]. For our network model, we have: 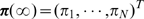 where 
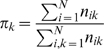
. The probability at the stationary state for residue 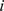 is the same as the fraction that the number of atomic contacts 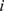 makes with other residues, plus the number of its non-interacting atoms, with the sum of all atomic contacts plus all non-interacting lone atoms in the whole structure (Supporting Information Text S1).

### Krylov Subspace Method

To analytically obtain the full dynamics response of residues in the PMT model, we discuss the Krylov subspace matrix reduction method for solving the master equation.

Noting the sparsity of the Markovian matrix 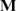, we follow the approach of Sidje [22]. Based on the analytical solution of matrix exponential shown in Eq. (3), one can expand 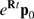 in the Krylov subspace 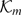, which is defined as:
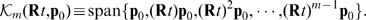
(4)


Denoting 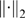 as the 2-norm of a vector or matrix, our approximation then becomes 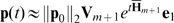, where 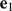 is the first unit basis vector, 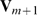 is a 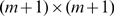 matrix formed by the orthonormal basis of the Krylov subspace, and 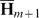 the upper Heisenberg matrix, both computed from an Arnoldi algorithm [41],[42]. The error can be bounded by 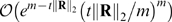. We now only need to compute explicitly the matrix exponential 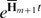. Because 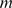 is much smaller than the total number of states, this is a simpler problem. A special form of the Padé rational of polynomials instead of Taylor expansion is used [22]: 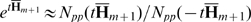, where 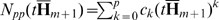 and 
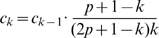
. All these calculations are done using Sidje's expokit software package [22]. We select 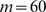 [22].

### Prediction Performance Assessment

We use sensitivity, specificity, and accuracy to assess the performance of our predictions in identifying functionally important residues. Sensitivity is defined as 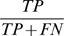, specificity as 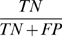, and accuracy as 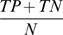, where 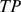, 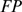, 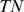, 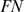, and 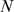 are the numbers of true positive, false positive, true negative, false negative, and total number of residues, respectively.

## Supporting Information

Video S1 Animation of Signal Transmission along Allosteric Pathway
(2.51 MB GIF)

Text S1 Biochemical and Structural Evidence of Predicted Key Residues, Stationary Distribution of the Perturbation-based Markovian Transmission Model, and Animation of Signal Transmission along Allosteric Pathway
(0.06 MB PDF)
