# Supplementary figures and images for: Perturbation-based Markovian Transmission Model for Probing Allosteric Dynamics of Large Macromolecular Assembling: A Study of GroEL-GroES
Source: PLoS Comput Biol. 2009 Oct 2;5(10):e1000526. doi: 10.1371/journal.pcbi.1000526 (PMC2741606; doi:10.1371/journal.pcbi.1000526)

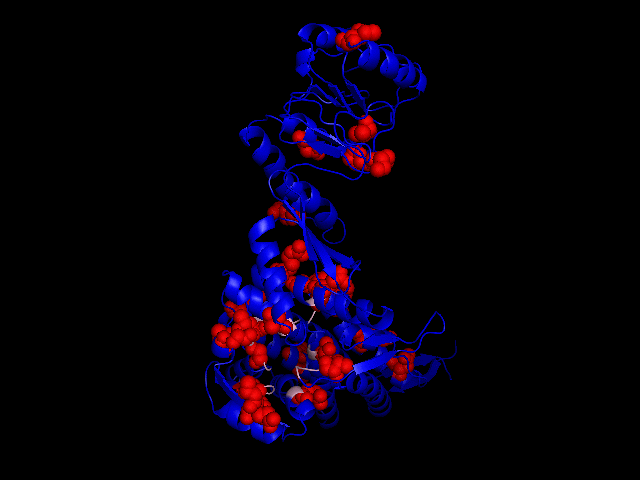

Supplement: Video S1 — Animation of Signal Transmission along Allosteric Pathway (2.51 MB GIF) [file pcbi.1000526.s001.gif]
