# Supplementary material for: Perturbation-based Markovian Transmission Model for Probing Allosteric Dynamics of Large Macromolecular Assembling: A Study of GroEL-GroES
Source: PLoS Comput Biol. 2009 Oct 2;5(10):e1000526. doi: 10.1371/journal.pcbi.1000526 (PMC2741606; doi:10.1371/journal.pcbi.1000526)
Supplement: Text S1 — Biochemical and Structural Evidence of Predicted Key Residues, Stationary Distribution of the Perturbation-based Markovian Transmission Model, and Animation of Signal Transmission along Allosteric Pathway (0.06 MB PDF) [file pcbi.1000526.s002.pdf]

Supporting Information Text S1

S1.1 Biochemical and Structural Evidence of Predicted Key Residues

Pivot residues

Pivot residues in the R" state. In the cis-ring chains of the R" state of GroEL, we have identified ten residues as pivot residues. These include the experimentally discovered pivot residue P137 [1]. The remaining nine residues (G45, A46, G110, G415, G431, G459, G471, G474, and P525) are located in the E-domains and are either Gly or Pro, which are special residues known to occur frequently at locations such as helix turns or helix bends. Among these predicted pivot residues for the R" state, five of them (G45, G110, G415, G431 and G471) are also predicted to be pivot residues in both R and T states. These residues are all located in the E-domain, experiencing least conformational changes among the three different states.

Residue G110 is located at the end of the relay helix D (residues 89-109). ATP binding in the cis-ring results in an offset across the ring-interface between the relay helix D on both cis- and trans-rings [1]. Upon ATP hydrolysis, helix D in cis-ring moves away from the ATP binding site towards the inter-ring interface [2]. G110 can be viewed as a pivot residue for this movement. A109 in cis-ring which is directly connected to G110 forms inter-ring contacts with another A109 from trans-ring [2]. It experiences one of the largest shift (3.8 Å) involved in cross-ring communication [1]. Additionally, G110 is reported to be involved in the interaction between cis-ring and trans-ring of GroEL [3].

G415 is located in a sharp helical turn, and forms a hydrogen bond with the ATP molecule [1]. It is likely that mutant in which a large residue replacing G145 may also lead to disfunction or reduced activity of chaperone. G431 is in the immediate neighborhood of E434, which is important for cross-ring communication [2]. E434 forms an inter-ring contacts with another E434 on a different chain in the trans-ring [2]. E434 in cis-ring also experiences one of the largest shift (4.9 Å) [1]. G431 therefore can be regarded as a pivot residue of this rearrangement.

The predicted pivot residue G459 in the R” state has a neighboring residue E461, whose mutation to K leads to loss of the ability of GroEL in aiding protein folding and the ability in releasing of the substrate protein [4]. It loses the inter-ring contact with R452 which exists in wild type, and makes contact instead with E434, and causes defective activity *in vitro* [2]. The quadruple mutant R452E/E461A/S463A/V464A is known to cause the dissociation of the two rings in GroEL [5].

Pivot residues in the R state. Additionally, there are nine residues predicted as pivot residues in the R state. Among them, two residues (A2 and G32) are in the E-domain, four (S139, T181, G182, and A384) are in the I-domain, and the remaining three (A243, G244 and G306) are in the A-domain.

In the E-domain, G32 is located at a helical turn, and forms a hydrogen bond with the phosphate of the ADP molecule [1]. It is likely that G32 plays an important role in ligand recognition of the nucleotide binding pocket.

In the I domain, the predicted pivot residue S139 is in the immediate neighborhood of residue C138. Mutant C138W is reported to cause loss of chaperone function [6]. For predicted pivot residues T181 and G182, T181 is located at a helical turn and maintains inter-domain contacts with F281 in the A domain in trans-ring [3]. Impaired pivot movement in F281D mutant leads to reduced ATPase activity and reduced ability to assist protein folding [4].

The predicted pivot residue A384 is adjacent to residue A383. Mutation of A383E leads to the loss of ATPase activity and the ability of GroES binding [4]. In addition, the neighboring residue E386 forms one salt-bridge with R197 in the T state and another salt bridge with K80 in the R state [7]. These salt bridges are important for the allosteric structural changes [8].

In the A-domain, predicted pivot residues A243 and G244 are immediate neighbors to the salt-bridge between K245 and E257 (on a different chain), which is formed during the transition between the T and R states [8], and is broken during the transition from the R to R” state [8]. Predicted pivot residue A243 has residue A241 on the potential substrate protein binding site [9]. Pivot residue G306 is involved in the interaction between cis-ring subunits in A-domain [3], and its immediate neighboring residue I305 has salt-bridge forming with residue A260 in the allosteric transition from R to R” state [8].

Pivot residues in the T state. In addition to pivot residues predicted from conformations in the R and R" state, there are five residues predicted as pivot residues for the T state conformation. Among them, three residues (S43, F44, and G86) are in the E-domain and the other two (T210 and G269) are in the A-domain.

In the E domain, predicted pivot residue S43 and F44 are adjacent to residue D41 and K42. Residue D41 makes an inter-subunit hydrogen bond with T522 to stabilize against possible pivoting movement in the T state [10, 11]. Additionally, K42 indirectly makes contact to D523 via the residue M267 in the A-domain [12]. Residue G86 is also predicted as a pivot residue, whose immediate neighbor D87 interacts Mg^{2+} ion of the ADP in the R" state to modulate possible conformational change during ADP molecules binding [1].

In the A-domain, the predicted pivot residue G269 has immediate neighbors, residue R268 and I270, that are reported as located in the region of substrate protein binding site [9]. They are likely also to participate in conformational change upon substrate binding.

Note that the dynamic responses of residues G375 and G410 reported as pivots in ref [1] are not the same as that of the other pivot residues P137 and G192, and are not classified as pivots by our definition. However, residues V376 and V411 directly neighboring these residues are predicted to be messenger residues. It is possible that residues G375 and G410 may play significant roles in transmitting signals between A-I and I-E domains, respectively, in addition to being the boundary points between different domains.

Messenger residues

Messenger residues in the R" state. In the cis-ring chains of the R" state conformation of GroEL, we are able to identify twenty four residues as messenger residues. Two (K245 and V273) are in the A-domain, and the remaining 22 residues are in the I-domain (A145, G148, A152-T157, G159, A163, A165, D167, V177, E186, K390, A394, V396, L400, A402, A406-E408).

In the A-domain, predicted messenger residue K245 forms a salt-bridge with residue E257 during the T-to-R transition. The salt-bridge is broken during the R-to-R" transition. This salt bridge plays important role in allosteric modulation of conformational changes [8]. Residue E257 is involved

in the binding of substrate protein [9]. Predicted messenger residue V273 in the A-domain neighbors V271, which is surface-exposed and has a distance of only 3.7 Å to the substrate protein, according to the structural study of [9]. It is likely that both V273 and V271 are important in transmitting signal for binding unfolded proteins.

Among the predicted messenger residue cluster of A152-T157 in the I-domain, the mutation of A152E, along with the I150E and S151V mutations are known to lead to the loss of ATPase activity and the loss of GroES binding ability for GroEL [4], suggesting the important functional roles these residues play in transmitting signal of conformational change. Structurally, S151 forms a hydrogen bond with D87, which interacts Mg^{2+} ion in the ADP binding pocket that coordinates the hydrolysis of ATP to ADP in the R" state [1]. The mutations to residues near S151 are likely to perturb the R87-Mg^{2+} interactions, resulting in the observed loss of ATPase activity. Without the conversion of ATP to ADP, GroEL cannot change conformations to that of the R state or the T state, resulting in arrested allosteric signal transmission. The predicted messenger residues V396 and L400 are also in the I domain, and are near residue D398, which also interacts with Mg^{2+} ion in the ADP binding pocket in the R" state [1]. Among the predicted messenger residue cluster of A406-E408, the mutations of A405E and A406E lead to the loss of the ATPase activities [4]. In addition, the predicted messenger residue E408 is an immediate neighbor of E409. The latter forms a salt bridge with R501, and is important for the inter-subunits interactions [13]. Mutation of E409A result in the stabilized T state relative to the R" state, leading to less effective allosteric communications [13].

The predicted messenger residues D155, A394, and V396, are involved in the D155-R395 salt bridge. It was shown that the single mutation of D155A breaks the interaction between D155 and R395, which in turn destroys the intra-ring symmetry, as less cooperative intermediates are generated that only switches conformations of a subset of the GroEL subunits upon the binding of ATP molecules [14]. Together with the study of unbiased molecular dynamic simulation by Sliozberg and Abrams [15], it was shown that the break of the salt bridge between D155 and R395 from the T state to the R state plays critical role in the positive cooperativity of the concerted allosteric transition.

Messenger residues in the T state. An additional 14 messenger residues are predicted from the T state structures: one of them is in the

A-domains (T294), and the remaining 13 are in the I-domains (S141, G146, V147, T149-S151, V376-K380, T385, and A399).

In the I-domains, among the predicted cluster of messenger residues T149-S151, mutations I150E and S151V, along with the mutation A152E to an immediate neighboring site all lead to the loss of the ATPase activity and the ability for GroEL to bind GroES [4]. Mutation A383E to the residue A383 that neighbors the two predicted messenger residues K380 and T385 leads to the loss of ATPase activity and the ability of GroES binding. This mutant chaperone is deficient in facilitating protein folding [4]. The predicted messenger residues T385 has immediate neighbors A384 and E386. Both of them make inter-subunit contacts between I-domain and A-domain. For example, A384 is in contact with Y360, and E386 is in contact with R197 and R285 [10]. In addition, residue E386 is an immediate neighbor of predicted messenger residue T385. It forms one salt bridge with R197 in the T state and another salt bridge with K80 in the R state [7]. These salt bridges are important for the allosteric structural changes [8, 16]. It is likely mutations to these predicted messenger will affect the function of GroEL. The predicted messenger residue A399 is an immediate neighbor of residue D398, which is located in the ATP binding pocket and forms hydrogen bond with Mg^{2+} in the R" state, which is critical in ATP hydrolysis to ADP [1].

Messenger residues in the R state. In the cis-ring chains of the R state conformation of GroEL, we are able to identify 24 messenger residues. Three of them are in the E-domains (V411, E460, and K498), and the other twenty one are in the A-domains (R197, N206, N265, T266, I270, V276, F281, R284-K286, L289, A293, T296, I342-G344, I353, A356, S358, V369, and K371).

In the E-domain, the predicted messenger V411 has a neighbor E409, which forms a salt bridge with R501, and is important for the inter-subunits interactions [13]. The predicted messenger residue E460 neighbors residue E461. Mutating it to E461K leads to loss of the ability of GroEL in aiding protein folding and reduce the ability of releasing substrate proteins [4]. In the neighborhood of the predicted messenger residue E460, several inter-ring contacts between residues in the E-domain of GroEL subunits have been found (G461:R452, S463:S463, and V464:V464) [10]. It suggests mutations on these residues may hinder the signal transmission between rings and destroy the stability of GroEL complex [16].

In the A-domain, the predicted messenger residue R197 is in contact with residue E386 in the I-domain of another chain [10]. This residue is also suggested to play important roles in passing signals between chains [16]. Residue F204 is a neighbor of the predicted messenger residue N206 in the A-domain. Mutation F204E leads to the loss of the ability for GroEL to bind GroES [4]. Mutating the predicted messenger N265 to Ala results in a GroEL molecule that is incapable of either binding GroES or assisting protein folding [4]. The predicted messenger residue I270 is found to be important in the binding of protein substrate [9]. Mutation K277 to I277 that neighbors the predicted messenger residue V276 results in the loss of the ATPase activity of GroEL [4]. Mutant F281D of the predicted messenger residue F281 loses ATPase activity and the ability assisting protein folding. Substrate protein aggregation is observed for this mutant [4]. Residue Q351 is a neighbor of the predicted messenger I353. This residue is important for the inter-subunits interactions in the A-domains of cis-ring in GroEL [3]. The predicted messenger S358 has immediate neighbors residues D359 and Y360; both of them form important contacts. D359 forms an salt bridge with K80 during the R to R" transition [8]. Y360 is in contact with A384, which is an inter-subunit contact between I-domain and A-domain [10].

Anchor residues

Anchor residues in the T state. In the cis-ring chains of the T state conformation of GroEL, we are able to identify twenty six residues predicted as anchor residues. There are fourteen residues in the E-domains (V27, V29, T30, A57, L62, T90, T91, L104, P462, V464, T482, I489, P496, and T517), five (V147, A152, V381, L400, and V407) in the I-domains, and the other seven (L222, E232, L248, A258, L262, V263, and V346) in the A-domains.

In the E-domain, D25 is a neighbor of the predicted anchor residue V27. Mutant D25R is found to reduce the ability of GroEL in assisting proteins folding [4]. The predicted anchor residue T30 is in the neighborhood of residue G32, which is located in the nucleotide binding pocket and forms a hydrogen bond with ADP in the R" state [1]. The predicted anchor residue A57 is adjacent to residue R58, which is identified as a member of the important network pathway for the allosteric communication in GroEL, based on a study of correlated mutations of GroEL [12]. The anchor residue T91 forms two hydrogen bonds with ADP in the the R" state [1].

Many mutational studies have been carried out around the predicted anchor residues P462 and V464, and the results often suggest the functional importance of these residues. For example, mutant E461K has reduced ability in assisting the folding and release of the substrate protein [4]. It loses the inter-ring contact with R452 as seen in wild type, and makes contact instead with E434, which causes defective activity *in vitro* [2]. The quadruple mutant R452E/E461A/S463A/V464A is known to cause the dissociation of the two rings in GroEL [5].

Neighboring to predicted anchor residue T482 and P496 in the E-domain, residue N479, A480, and D495 are located in the ATP binding pocket and all form hydrogen bonds with the ADP in the R" state [1]. C519 neighbors the predicted anchor residue T517. The mutant C519S destroys the inter-chain contact between the subunits in GroEL, resulting in partial dissociation of the GroEL subunits [2, 17].

In the I-domains, the mutations I150E, A152E and A383E of residues neighboring the anchor residues A152 and V381 all experience loss of ATPase activity, ability of binding GroES, assisting protein folding, and releasing substrate protein [4]. In the neighborhood of anchor residue L400, residue D398 is located in the ATP binding pocket of the GroEL subunits. It interacts with Mg^{2+}, which in turn assists the hydrolysis of ATP to ADP in the R" state [1]. Mutant of A406E, which is immediately connected to the predicted anchor residue V407, loses its ATPase activity and GroES binding ability, with reduced function of substrate binding and folding [4].

In the A-domain, residue I230 and L234 are neighbors of the predicted anchor residue E232. They are found to be important in the binding of protein substrate [9]. Mutant L234E has been shown to decrease the ATPase activity of GroEL. This mutant cannot bind to GroES and substrate proteins [4].

Predicted anchor residue A258 is an immediate neighbor to residue E257 and L259. E257 senses substrate protein binding and its mutation to Ala leads to the loss of the ability in detecting substrate protein. This mutant is not capable of stimulating the ATPase activity of GroEL, hence cannot initiate the allosteric communications [7]. E257 is also found to have hydrogen-bonded interaction with substrate peptide [9]. Residue L259 has been suggested to play the role of binding substrate proteins. The mutation L259S leads to the loss of the ability of GroEL to bind both GroES and

substrate protein [4, 9].

Predicted anchor residues T261 and V263 has been found to have important hydrophobic interaction with the substrate proteins [9]. Mutant V263S is observed to be deficient in binding substrate proteins [4].

Anchor residues in the R state. In addition to residue A57, L104, P462, V464, and T482 predicted as anchor residues in the T states, we are able to identify additional twenty one anchor residues in the cis-ring chains of GroEL subunits in the R state. There are eleven residues in the E-domains (L40, T89, M111, A405, L419, C458, A466, M491, V499, V510, and A511), three are in the I-domains (K142, I150, and V190), and the other seven are in the A-domains (L200, L215, I230, I250, E252, V273, and Q343).

In the E-domains, residue T91 is one of the neighbors of anchor residue T89. It forms two hydrogen bonds with ADP in the the R" state [1]. Mutants D87N and D87K both are observed to have lost ATPase activity, the ability of assisting and releasing substrate protein folding, and have reduced GroES binding ability [4]. The mutant A405E of the predicted anchor residue A405 is observed to have similarly lost ATPase activity, the ability to assist folding, and the ability in releasing substrate protein [4]. In the neighborhood of the predicted anchor residue A466, the quadruple mutant R452E/E461A/S463A/V464A is known to cause the dissociation of the two rings in GroEL [5]. The neighbor R501 of the predicted residue V499 forms a salt bridge with E409, and is important for the inter-subunits interactions [13].

In the I-domains, the mutant I150E of the predicted anchor residue T150 is observed to have lost ATPase activity, the ability of binding GroES, the ability to assist protein folding, and has reduced release of substrate protein [4].

In the A-domain, residue S201 and Y199 are immediately connected to the predicted anchor residue L200. Mutant S201E is known to have reduced ability of substrate protein binding and folding [4]. Residue Y199 is found to have interaction with unfolded polypeptide [9], and its mutant Y199E is observed to have lost the ability to bind GroES and substrate proteins [4]. The predicted anchor residue I230 is also found to have interaction with unfolded polypeptide [9]. Predicted anchor residue V273 in the A-domain neighbors V271, which is surface-exposed and has a distance of only 3.7 Å to the substrate protein [9].

Anchor residues in the R" state. We are able to further identify additional twenty one anchor residues in the cis-ring chains of GroEL subunits in the R state. There are seventeen residues in the E-domains (N37, V38, T50, D52, S55, S79, A81, E129, K425, E483, I493-D495, S509, I515, T516, and C519) and the other four are in the A-domains (I205, N206, A212, and D328).

In the E-domains, the predicted anchor residues T50, D52, and S55 are in the neighborhood of residues T50 and V54, which are found to be relatively conserved based on sequence analysis [18]. Anchor residues S79 and A81 are immediately connected to K80, and the latter forms a salt-bridge with E386 in the R state conformation [19]. This salt bridge is involved in the rotation of the I-domain of GroEL subunits by switching inter-subunit contacts from its neighboring A-domain to the E-domain upon binding of ATPs [19]. Among anchor residues I493-D495, D495 is located on the surface of the nucleotide binding pocket and plays an important role in ADP binding through hydrogen bond with ADP in the R" state [1]. By destroying the inter-subunit contact of GroEL, the mutant C519S of the anchor residue C519 partially dissociates [2]. In addition, the interaction between C137 and C518 is involved in the release of the substrate protein [17].

In the A-domains, the predicted anchor residues I205 and N206 are adjacent to the residue F204. Mutant F204E is located on the substrate protein binding surface and is important for the binding of GroES and substrate protein [4]. F204 is also involving the interaction of cis-ring in A domain of GroEL [3]. The predicted anchor residue D328 is one of the network residues involved in the inter-subunit interaction of GroEL with R58 and A81 [12].

S1.2 Stationary Distribution of the Perturbation-based Markovian Transmission Model

The Markovian transition matrix $\boldsymbol{M} = \{m_{ij}\}$, with elements $m_{ij} = 0$ if there is no atom-atom contacts between residue i and j, and $m_{ij} = \frac{n_{ij}}{\sum_i n_{ij}}$ if there are atom-atom contacts between residue i and j. This stochastic Matrix has an eigenvalue of 1, and there exists a column vector $\boldsymbol{x} > 0$ that satisfies:

$$\boldsymbol{M}\boldsymbol{x} = \boldsymbol{x}.$$

Since $n_{ij} = n_{ji}$ for $i \neq j$, if we let $x_k = \sum_{i=1}^{N} n_{ik} = \sum_{j=1}^{N} n_{kj}$, N being the total number of residues, the column vector $\boldsymbol{x}$ satisfies the following equation:

$$
\begin{aligned}
\boldsymbol{Mx} &= (\sum_{j=1}^{N} m_{1j}x_j, \sum_{j=1}^{N} m_{2j}x_j, \cdots, \sum_{j=1}^{N} m_{Nj}x_j)^T \\
&= (\sum_{j=1}^{N}(\frac{n_{1j}}{\sum_{i=1}^{N} n_{ij}} \sum_{i=1}^{N} n_{ij}), \sum_{j=1}^{N}(\frac{n_{2j}}{\sum_{i=1}^{N} n_{ij}} \sum_{i=1}^{N} n_{ij}) \cdots, \sum_{j=1}^{N}(\frac{n_{Nj}}{\sum_{i=1}^{N} n_{ij}} \sum_{i=1}^{N} n_{ij}))^T \\
&= (\sum_{j=1}^{N} n_{1j}, \sum_{j=1}^{N} n_{2j}, \cdots, \sum_{j=1}^{N} n_{Nj})^T \\
&= (\sum_{i=1}^{N} n_{i1}, \sum_{i=1}^{N} n_{i2}, \cdots, , \sum_{i=1}^{N} n_{iN})^T \\
&= (x_1, x_2, ..., x_N)^T \\
&= \boldsymbol{x}.
\end{aligned} \tag{1}
$$

That is, $\boldsymbol{x}$ as written is proportional to the vector of stationary distribution $\boldsymbol{\pi}$, which satisfies $\boldsymbol{\pi} = \boldsymbol{M\pi}$. Normalizing $\boldsymbol{x}$, we obtain the stationary distribution:

$$\boldsymbol{\pi} = (\pi_1, \ldots, \pi_N),$$

where

$$\pi_k = \frac{x_k}{\sum_{k=1}^{N} x_k} = \frac{\sum_{i=1}^{N} n_{ik}}{\sum_{k=1}^{N}\sum_{i=1}^{N} n_{ik}}.$$

S1.3 Animation of Signal Transmission along Allosteric Pathway

In order to study the allosteric signaling transmission pathway of the GroEL-GroES complex, we apply uniform perturbation on residues located in the nucleotide binding pocket only and record the dynamic responses of all residues in the cis-ring of the GroEL-GroES chaperone complex. Since there are multiple symmetrically related identical chains in each ring, we take the average

values of the time-dependent probability flow for residues in multiple chains from the same ring.

As shown in the movie (Vedio S1), the amount of probability flow at each residue changes with time. The temporal change of the probability flow is visualized in log time scale. The value of the probability flow from low to high is colored from blue to red. Initially, the following residues are perturbed: N479, A480, G415, D495, G32, T91, D398, D87, S151, L31, I454, I150, I493, P33, and A481, which either form H-bond or have van der Waals interaction with the ADP molecules [1] (shown in light pink color).

Based on our calculations, there are 28 residues which can be selected as on-pathway residues, and they are: T30, N68, K75, K80, D83, G86, T90, D115, R118, K132, T149, I270, V276, P279, D291, K327, E354, E397, H401, R404, G414, Q453, Y478, T482, E483, I489, G492, and L494. These residues are shown in red color in space-filling model.

References

1. Xu Z, Horwich A, Sigler P (1997) The crystal structure of the asymmetric GroEL-GroES-(ADP)7 chaperonin complex. Nature 388(6644):741–50.

2. Ranson NA, Clare DK, Farr GW, Houldershaw D, Horwich AL, et al. (2006) Allosteric signaling of ATP hydrolysis in GroEL-GroES complexes. Nature Structural and Molecular Biology 13:147–152.

3. Zheng W, Brooks B, Thirumalai D (2007) Allosteric transitions in the chaperonin GroEL are captured by a dominant normal mode that is most robust to sequence variations. Biophys J 93:2289–99.

4. Fenton W, Kashi Y, Furtak K, Horwich A (1994) Residues in chaperonin GroEL required for polypeptide binding and release. Nature 371:614–9.

5. Weissman JS, Hohl CM, Kovalenko O, Kashi Y, Chen S, et al. (1995) Mechanism of GroEL action: Productive release of polypeptide from a sequestered position under GroES. Cell 83:577–587.

6. Miyazaki T, Yoshimi T, Furutsu Y, Hongo K, Mizobata T, et al. (2002) GroEL-substrate-GroES ternary complexes are an important transient intermediate of the chaperonin cycle. Journal of Biological Chemistry 277:50621–50628.

7. Danziger O, Shimon L, Horovitz A (2006) Glu257 in GroEL is a sensor involved in coupling polypeptide substrate binding to stimulation of ATP hydrolysis. Protein Science 15:1270–1276.

8. Hyeon C, Lorimer G, Thirumalai D (2006) Dynamics of allosteric transitions in GroEL. Biophys J 103(50):18939–44.

9. Buckle AM, Zahn R, Fersht AR (1997) A structural model for GroEL-polypeptide recognition. Proceedings of the National Academy of Sciences of the United States of America 94:3571–3575.

10. Braig K, Otwinowski Z, Hegde R, Boisvert DC, Joachimiak A, et al. (1994) The crystal structure of the bacterial chaperonin GroEL at 2.8 Å. Nature 371:578–586.

11. Braig K, Adams PD, Brunger AT (1995) Conformational variability in the refined structure of the chaperonin GroEL at 2.8 Å resolution. Nature Structural Biology 2:1083–1094.

12. Kass I, Horovitz A (2002) Mapping pathways of allosteric communication in GroEL by analysis of correlated mutations. Proteins: Structure, Function and Genetics 48:611–617.

13. Aharoni A, Horovitz A (1997) Detection of changes in pairwise interactions during allosteric transitions: Coupling between local and global conformational changes in GroEL. Proceedings of the National Academy of Sciences of the United States of America 94:1698–1702.

14. Danziger O, Rivenzon-Segal D, Wolf S, Horovitz A (2003) Conversion of the allosteric transition of GroEL from concerted to sequential by the single mutation Asp-155-Ala. Proc Natl Acad Sci U S A 100:13797–13802.

15. Sliozberg Y, Abrams C (2007) Spontaneous conformational changes in the e. coli GroEL subunit from all-atom molecular dynamics simulations. Biophys J 93:1906–1916.

16. Chennubhotla C, Bahar I (2006) Markov propagation of allosteric effects in biomolecular systems: application to GroEL-GroES. Mol Syst Biol 2:36.

17. Horovitz A, Bochkareva ES, Yifrach O, Girshovich AS (1994) Prediction of an inter-residue interaction in the chaperonin GroEL from multiple sequence alignment is confirmed by double-mutant cycle analysis. Journal of Molecular Biology 238:133–138.

18. Stan G, Thirumalai D, Lorimer GH, Brooks BR (2003) Annealing function of GroEL: Structural and bioinformatic analysis. Biophysical Chemistry 100:453–467.

19. Ranson NA, Farr GW, Roseman AM, Gowen B, Fenton WA, et al. (2001) ATP-bound states of GroEL captured by cryo-electron microscopy. Cell 107:869–879.
